# Identification of *Theileria lestoquardi* Antigens Recognized by CD8^+^ T Cells

**DOI:** 10.1371/journal.pone.0162571

**Published:** 2016-09-09

**Authors:** Shan Goh, Daniel Ngugi, Regina Lizundia, Isabel Hostettler, Kerry Woods, Keith Ballingall, Niall D. MacHugh, W. Ivan Morrison, Willie Weir, Brian Shiels, Dirk Werling

**Affiliations:** 1 Department of Pathology and Pathogen Biology, Royal Veterinary College, Hawkshead Lane, Hatfield, AL9 7TA, United Kingdom; 2 Institute of Parasitology, Vetsuisse Faculty, University of Bern, Laenggassstrasse 122, 3012 Bern, Switzerland; 3 Institute of Animal Pathology, Vetsuisse Faculty, University of Bern, Laenggassstrasse 122, 3012 Bern, Switzerland; 4 Moredun Research Institute, Pentlands Science Park, Bush Loan, Penicuik, Midlothian, EH26 0PZ, United Kingdom; 5 The Roslin Institute, Royal (Dick) School of Veterinary Studies, University of Edinburgh, Easter Bush, Midlothian, EH25 9RG, United Kingdom; 6 Institute of Biodiversity Animal Health and Comparative Medicine, College of Medical, Veterinary and Life Sciences, University of Glasgow, Bearsden Road, Glasgow, G61 1QH, United Kingdom; Institut national de la santé et de la recherche médicale—Institut Cochin, FRANCE

## Abstract

As part of an international effort to develop vaccines for *Theileria lestoquardi*, we undertook a limited screen to test *T*. *lestoquardi* orthologues of antigens recognised by CD8^+^ T lymphocyte responses against *T*. *annulata* and *T*. *parva* in cattle. Five MHC defined sheep were immunized by live *T*. *lestoquardi* infection and their CD8^+^ T lymphocyte responses determined. Thirteen *T*. *lestoquardi* orthologues of *T*. *parva* and *T*. *annulata* genes, previously shown to be targets of CD8^+^ T lymphocyte responses of immune cattle, were expressed in autologous fibroblasts and screened for T cell recognition using an IFNγ assay. Genes encoding *T*. *lestoquardi* antigens Tl8 (putative cysteine proteinase, 349 aa) or Tl9 (hypothetical secreted protein, 293 aa) were recognise by T cells from one animal that displayed a unique MHC class I genotype. Antigenic 9-mer peptide epitopes of Tl8 and Tl9 were identified through peptide scans using CD8^+^ T cells from the responding animal. These experiments identify the first *T*. *lestoquardi* antigens recognised by CD8^+^ T cell responses linked to specific MHC class I alleles.

## Introduction

*Theileria* species are tick-transmitted hemoprotozoan parasites infecting wild and domestic ungulates in many areas of the world. The most economically important species are *T*. *parva* and *T*. *annulata*, which are pathogenic to cattle, and *T*. *lestoquardi* (formerly *T*. *hirci*), which is pathogenic to sheep. Sheep are valuable commodities in North Africa, Asia, and the Middle East, and there is a need for better prevention and/or treatment measures in order to reduce the economic burden of disease caused by *T*. *lestoquardi*.

Infection with *T*. *lestoquardi* causes an acute disease known as malignant ovine theileriosis. Clinical signs include loss of condition, coughing, lethargy, enlargement of lymph nodes, and fever [1]. Mortality levels of up to 73% have been reported for malignant ovine theileriosis [2] and the disease can lead to reduced productivity and spontaneous abortions in survivors [3]. *T*. *lestoquardi* is transmitted by *Hyalomma anatolicum* ticks [4] and possibly other *Hyalomma* spp. [5, 6] and, as with most vector-borne diseases, control of transmission is challenging [7]. In addition, current disease control methods are limited to treatment with a theilericidal compound, buparvaquone [8, 9], and in some countries, vaccination with attenuated parasitized cell lines [10–12]. These methods are not easy to apply successfully. Preparation of attenuated *T*. *lestoquardi-* infected cell line vaccines suitable for vaccination requires prolonged *in vitro* passage and thorough testing *in vivo*, and distribution of the vaccine is dependent on a cold chain. There are also issues with quality control, reproducibility and potential reversion to virulence [13, 14]. Furthermore, the mechanisms of attenuation are only partially understood [14–16]. A subunit vaccine would circumvent the logistical constraints of attenuated cell line vaccines. However, development of a subunit vaccine requires a greater understanding of protective immune responses against *T*. *lestoquardi* and the antigens that they recognise.

Studies of immune responses to, and antigen identification in, *T*. *lestoquardi* are lagging behind those for *T*. *parva* and *T*. *annulata* for which antigens were identified through screening of random *T*. *parva* and selected *T*. *annulata* and *T*. *parva* schizont cDNA clones with potential to transform host cells [17–19]. Although the disease produced by these parasites and the immune responses they induce are very similar, evidence of genetic and antigenic similarities are most evident for *T*. *lestoquardi* and *T*. *annulata*. These include cross-reactivity of *T*. *lestoquardi* antisera with *T*. *annulata* antigens [20], serological identification of *T*. *lestoquardi* proteins with amino acid sequence similarity to *T*. *annulata* and *T*. *parva* [21, 22], infection of similar cell types by *T*. *lestoquardi* and *T*. *annulata* in sheep and cattle respectively [23], and the higher similarity between *T*. *lestoquardi* and *T*. *annulata* 18S rRNA sequences [24]. There have been no reports on cellular responses to antigens conserved across *T*. *lestoquardi*, *T*. *annulata* and *T*. *parva*; but these may occur given the identification of a conserved T cell antigen between *T*. *annulata* and *T*. *parva* [19].

There is evidence that immunity to *T*. *parva* and *T*. *annulata* in cattle involves T cell mediated responses; both CD4^+^ and CD8^+^ T cells recognise parasitized leukocytes [25, 26]. An important role for CD8^+^ T cell responses in protection against *T*. *parva* has been demonstrated by adoptive transfer of immune CD8^+^ T cells [27], and it has been proposed that help from CD4^+^ T cells may also be required [28]. CD8^+^ T cell responses to *T*. *parva* and *T*. *annulata* antigens are MHC class I restricted [29, 30], and in individual animals the responses are frequently focused on a few immunodominant antigens, which differ depending on the MHC genotype of the animal [19, 31].

Based on genetic and pathogenic similarities of *T*. *lestoquardi* to *T*. *annulata* and *T*. *parva*, we propose that similar responses are likely to be involved in immunity against *T*. *lestoquardi* and that recognition of immunodominant T cell antigens orthologous to those of *T*. *annulata*/*T*. *parva* may occur. This study, therefore, aimed to obtain evidence of induction of CD8^+^ T cell responses by *T*. *lestoquardi* and to identify parasite antigens recognised by the specific CD8^+^ T cell response based on screening orthologues of those recognised in *T*. *annulata* and *T*. *parva*.

## Methods

### Ethics statement

Animal care and use were approved by the Royal Veterinary College Ethics Committee with the Home Office Project licence number PPL 60/3736. Animal work was carried out in accordance with the UK government Animals (Scientific Procedures) Act (ASPA) 1986.

### Animals

Five adult Swaledale/Leicester cross sheep (approximately 4 years old) were used in this study. Animals were euthanized at the end of the study with lethal injection of barbituates.

### Immunization

Sheep were immunized by subcutaneous administration of 1–3 × 10^6^ cells of the *T*. *lestoquardi*-infected cell line, THS1 [32], as previously described [11]. Sheep were treated with buparvaquone (2.5mg kg^-1^, Bimeda, Ireland) if fever persisted for two consecutive days. One week after recovery from clinical reactions, sheep were re-challenged with the same number of autologous parasitized cells without treatment to confirm and boost their immunity. Health of the sheep was monitored by daily measurement of rectal temperature and palpation of the draining lymph node.

### MHC class I and II genotyping

MHC class I genotyping was carried out as previously described [33] with some modifications. Total RNA was extracted with the Qiagen RNeasy Mini kit from cryopreserved lymphocytes of *T*. *lestoquardi*-infected sheep. Contaminating DNA was removed with the Turbo DNA-free™ kit (Applied Biosystems) before cDNA synthesis, which was carried out using the AMV LongAmp® Taq RT-PCR kit (New England Biolabs). Partial MHC class I sequences were amplified from cDNA samples using class I generic primers 416 (5' CGGCTACGTGGACGACAYG 3') and Cr (5' ATGGGTCACATGTGYCTTTG 3'), which bind within exons 1 and 3 [33], generating a 500 bp product. The cycling conditions for PCR were 94°C for 4 min, 30 cycles 94°C for 30 s, 55°C for 30 s, and 72°C for 30 s, with Promega Go *Taq* DNA polymerase. Amplicons were gel purified (QIAquick Gel Extraction kit, Qiagen) and Sanger sequenced (Eurofins UK) using the same primer set, resulting in multiple sequences of different alleles. Individual class I sequences were determined by cloning the purified amplicons into pGEM®-T Easy vector (Promega). Thirty transformants per sample were selected for bidirectional Sanger sequencing with vector-specific T7 and SP6 primers. Class I sequences were aligned using the SeqMan Pro programme within the DNASTAR Lasergene 11 package, and a consensus sequences of each allele was generated from a minimum of 3 independent clones. Each consensus allelic sequence was BLASTN searched against an in house database of known ovine class I sequences. Novel alleles were added to this database, and assigned a unique name that reflects the order in which they were identified. MHC class II genotyping was carried out as previously described [34]. The novel class II *DRB1* sequence was validated by cloning the complete second exon sequence. The sequence was submitted to the ENA and IPD-MHC database for an official allelic nomenclature.

### Generation of *T*. *lestoquardi* infected cell lines

*T*. *lestoquardi*-infected cell lines were obtained from peripheral blood or lymph nodes of infected sheep. Blood or lymph node aspirates taken from the draining lymph node nearest the site of challenge were collected daily in Alsever’s solution (Sigma Aldrich), from day 12 post-infection but before treatment with buparvaquone [35]. PBMC and lymph node mononuclear cells were separated from the blood or lymph node aspirate using Ficoll-Paque (GE Life Sciences) according to the manufacturer’s recommendations, and resuspended in culture medium (RPMI 1640 Glutamax medium, Life Technologies, Gibco, Paisley, UK) supplemented with 10% FCS (GIBCO), penicillin-streptomycin (5000 units ml^-1^ and 5 mg ml^-1^, respectively, Sigma-Aldrich, Dorset, UK) and 50 μM of 2-mercaptoethanol (Sigma Aldrich). PBMC and/or lymph node cells were counted and dispensed at 2.5 × 106–1 × 10^7^ cells per well in 2 ml culture medium in 24 well plates and cultured until parasitized cell lines were established [35]. The cultures were incubated at 37°C in 5% CO_2_.

### Cloning of candidate *T*. *lestoquardi* genes

Total RNA was extracted from *T*. *lestoquardi*-infected PBMC of the study sheep, using either the RNeasy Mini Kit (Qiagen) or Trizol (Invitrogen), and cDNA was prepared using either AMV First strand cDNA synthesis kit (New England Biolabs) or QuantiTect Reverse Transcription kit (Qiagen). PCR was carried out either with Phusion Mastermix (Thermo Scientific) or *Pfu* DNA polymerase (Promega), and *T*. *lestoquardi* gene-specific primers were used for amplification of full-length cDNA (S1 Table). An exception was antigen Tl12, which was amplified from genomic DNA of *T*. *lestoquardi* infected PBMC. Genomic DNA was extracted using the DNeasy Blood and Tissue kit (Qiagen). Amplicons were cloned into a modified pMax expression vector (Lonza), containing a C-terminal V5-tag, by restriction-ligation. Exceptions were Tl2, Tl13245 (orthologue of *T*. *annulata* TA13245), and Tl16020 (orthologue of *T*. *annulata* TA16020), which were first cloned into pJET1.2 (Thermo Scientific) for gene amplification and then cloned into pMax either by restriction-ligation, or by sequence and ligation-independent cloning (SLIC) [36] (S1 Table). Briefly, SLIC involved linearization of pMax either by digestion with EcoRV and ScaI (Fermentas), or by inverse PCR with primers pMax_EcoRV and pMax_XhoI (S1 Table). Insert amplicons, generated from primers containing gene and vector-specific sequences, were purified and mixed with linearized vector, 0.6–1.5 U T4 DNA polymerase (New England Biolabs), 1 × BSA, and 1 × NEB Buffer 2 for 2.5 min. Recombinant plasmids of Tl1, 2, 3, 5, 6, 7, 8, 9, 10, 12, 16, 13245, or 16020 were transformed in either DH5α or DH10B cells (NEB, UK) and selected on LB plates supplemented with kanamycin (50 μg ml^-1^). Recombinant plasmids were Sanger sequenced to validate insert identity.

### Culture of sheep skin fibroblast

Skin biopsies of approximately 1 cm^2^ were taken from immunized sheep to establish fibroblast cell lines with matching MHC to effector cells from the same sheep. Skin biopsies were washed in culture medium (RPMI with 10% FCS, and penicillin (5000 units ml^-1^)-streptomycin (5 mg ml^-1^)), cut into small pieces, adhered onto culture dishes mechanically, and air-dried for 1 h. Skin was then covered with culture media and incubated at 37°C in 5% CO_2_ for 1–3 weeks, or until fibroblast grew onto plastic wells. Culture media was changed weekly. Fibroblast cell lines were passaged every 3–4 days or as they reached confluency.

### Transfection of ovine fibroblasts with *T*. *lestoquardi* antigenic genes

Plasmids for transfections were prepared from a single batch of 25 ml bacterial cultures using the Qiagen Plasmid Midi kit. Autologous fibroblast cells were seeded in 96 well plates at 1 × 10^4^ per well in 100 μl of culture medium and incubated overnight at 37°C in 5% CO_2_. Transfection was carried out either with Lipofectamine LTX & Plus Reagent (Invitrogen) or Fugene (Promega) at reagent (μl): DNA (μg) ratios of 5:1 and 3:1, respectively. Lipofectamine transfected fibroblasts at 24 h and 48 h were examined for V5 tag expression by immunofluorescence as described previously [37]. Briefly, fibroblasts were seeded at 5 × 10^4^ per well in 1 ml culture medium on glass coverslips in 24 well plates and incubated overnight at 37°C in 5% CO_2_. Cells were transfected either for 24 h or 48 h, fixed with 4% paraformaldehyde (Sigma Aldrich) and permeabilized with 0.2% Triton-X (Sigma Aldrich) for 10 min each. Cells were then blocked with 10% FCS for 30 min before incubation with anti-V5 mouse antibody (diluted 1:500, Invitrogen) for 1 h, followed by incubation with an anti-mouse goat antibody AlexaFluor 488 (diluted 1:1500, Invitrogen). Cells were counter-stained with 1 μM DAPI (Life Technologies) for 10 min, coverslips were blotted dry and mounted onto glass slides for microscopy with DAKO fluorescence mounting medium (Agilent Technologies). Fluorescence microscopy was carried out using an Olympus BX60 at 1000 × magnification, using appropriate filter cubes and the CoolLED pE excitation system (Nikon, UK). Images were captured using the Image-Pro Plus 5.0 (MediaCybernetics).

### Stimulation and enrichment of CD8^+^ T cells

CD8^+^ T cells were prepared as previously described with slight modifications [38]. PBMC (effectors) were collected from immunized sheep at 28 days post immunization. Gamma irradiated (100 Gy) autologous *T*. *lestoquardi* infected cell lines generated as described above were used as stimulators. Effectors and stimulators were co-cultured at an effector to stimulator ratio of 20:1 in 2 ml of culture medium in 24 well plates, and incubated at 37°C for one week in 5% CO_2_. Cells were then harvested and dead cells removed using Ficoll-Paque (GE Life Sciences). Viable effector cells were re-stimulated again at an effector to stimulator ratio of 10:1. After an additional week, cells were harvested, separated on Ficoll-Paque, and enriched for CD8 by complement fixation to remove CD4, γδ T cells and NK T cells. To do so, a cocktail of the following mouse-anti sheep or bovine antibodies, each at 8 μg ml^-1^, was used: anti-CD4^+^ T cells (MCA2213GA; AbD Serotec), anti-γδ T cells (MCA838G; AbD Serotec), anti-NK T cells (MCA5933GA; AbD Serotec). The cocktail was added to 5 × 10^7^ ml^-1^ effector cell suspension in equal volumes, for a final concentration of 4 μg ml^-1^ per antibody. The mixture was incubated on ice for 30 min for opsonisation to take place. One part of rabbit serum was added to two parts of the cells/antibody mixture and incubated at 37°C for 45 min. Thereafter, cells were washed in 10 ml culture medium, rested at 37°C for 2–3 h, spun on Ficoll-Paque to remove debris and harvested cells plated at 5 × 10^3^ per well in a 96 well plate. Cells were re-stimulated with 1 × 10^3^ irradiated *T*. *lestoquardi* cells in the presence of 100 U ml^-1^ of recombinant human (rhu) IL-2 (Proleukin®, Novartis). The cultures were incubated at 37°C in 5% CO_2_ and used as effectors in an IFNγ production assay 14–21 days later.

### Phenotyping of enriched T cells

The phenotype of effector T cell populations was determined by FACS analysis using antibodies described above, as well as mouse- antisheep or antibovine MHC class I (MCA2444GA; AbD Serotec), B-cell (MCA2443GA; AbD Serotec) and CD8 (MCA2216GA; AbD Serotec). Cells (2 × 10^5^–1 × 10^6^) were mixed with an equal volume of primary antibody (final concentration 1 μg ml^-1^), incubated at 4°C for 30 min, washed three times using PBS, and resuspended in 50 μl FACS medium (2% horse serum in PBS). FITC-labelled goat anti-mouse IgG (AbD Serotec) was used as secondary antibody. Cells were incubated at 4°C for 30 min, washed as before and resuspended in FACSFlow (Becton Dickinson Biosciences) for data acquisition and analysis using either a FACSCalibur (Becton Dickinson Biosciences) or a FACSAria (Becton Dickinson Biosciences).

### Cytotoxicity assay

*T*. *lestoquardi* infected cell lines (targets) were labelled with Indium oxine (^111^In) (GE Healthcare UK) by incubating 50 μl (1 × 10^6^) cells with 0.185 Mbq of ^111^In for 15 min at 37°C. Labelled target cells were washed six times in washing medium (RPMI 1640 Glutamax medium with 2% FCS) then resuspended in culture medium. Effector cells, either in stimulated whole PBMC or stimulated and enriched CD8^+^ T cells prepared with *T*. *lestoquardi*-infected cell lines as described above, were mixed with labelled target cells in duplicates in two-fold dilutions starting at 80:1 in a final volume of 150 μl. Positive controls consisted of labelled target cells lysed with 100 μl of 0.2% Tween20, negative controls were unlabelled target cells, and all controls were performed in triplicate. Cells were incubated for 4 h at 37°C, resuspended in the same volume of culture medium and centrifuged at 250 × g for 10 min. Seventy-five μl of supernatant from each sample was measured for radioactivity in a gamma counter (WALLAC 1470, Perkin Elmer). Percentage cytotoxicity was calculated as release of ^111^In according to 100 × (test release–medium release) / (Tween20 release–medium release).

### IFNγ ELISA

Fibroblasts transfected with plasmid DNA or treated with peptides overnight were washed three times before addition of 2.5 × 10^5^ effector cells per well in 200 μl culture medium, and incubated for 72 h at 37°C in 5% CO_2_. Cell supernatants were harvested and analysed by IFNγ ELISA, according manufacturer’s instruction (MABTECH^TM^); ELISA reactions were recorded on a SpectraMax M2 (Molecular Devices) plate reader. All assays included the following controls: culture medium only, fibroblast only, effectors only, effectors and stimulators, fibroblast and stimulators, fibroblast transfected with plasmid expressing GFP, and for peptide assays—fibroblasts transfected with plasmid expressing Tl8 or Tl9.

### Peptide library designs

Peptides derived from the amino acid sequences of Tl8 and Tl9 were synthesized as 17-mers with 12 aa overlapping (JPT Peptide Technologies, Germany). Sixty-eight Tl8 peptides and 57 Tl9 peptides were tested. Peptides (approx. 67 nmol) were resuspended in RPMI in a 96 well format. Up to 8 peptides in the same row were pooled, and up to 10 peptides in the same column were pooled so that each peptide was present in both row and column pools and used for IFNγ assay. Peptide pools (1 μg ml^-1^ per peptide) were incubated with fibroblasts in 200 μl culture medium for IFNγ ELISA, and putative positive peptides were confirmed by peptide titration (0.01–10 μg ml^-1^) in the IFNγ assay. T cell epitope sequences were further examined by synthesizing 9–12-mer peptides of each epitope truncated sequentially either at the N or C terminal (JPT Peptide Technologies, Germany). Peptides were dissolved in up to 20% DMSO in PBS to 10 mg ml^-1^, then diluted in RPMI for IFNγ assays at 1–10 μg ml^-1^ as above.

### Nucleotide sequences

Ovine MHC class I and II allele sequences were deposited in the European Nucleotide Archive with accession numbers LN868342 –LN868359 and HF954377 (Table 1). *T*. *lestoquardi* gene sequences were deposited in Genbank with accession numbers KT989585—KT989597 (Table 2).

**Table 1:** MHC class I and II alleles identified for individual sheep.

Animal	Class I sequences (Accession number)^a^	Class II DRB (Accession number)	Reference
1263	Ovar-I*2K (LN868358), Ovar-I*U (LN868342), Ovar-I*2L (LN868359)	Ovar-DRB1*1802 homozygous (HF954377)	This study
1343	Ovar-I*V (LN868343), Ovar-I*W (LN868344), Ovar-I*X (LN868345)	Ovar-DRB1*0801, Ovar-DRB1*1201	[39]
1360	Ovar-I*U (LN868342), Ovar-I*V (LN868343), Ovar-I*Y (LN868346), Ovar-I*Z (LN868347)	Ovar-DRB1*0501 homozygous	[39]
4223	Ovar-I*2A (LN868348), Ovar-I*2B (LN868349), Ovar-I*2C (LN868350), Ovar-I*2D (LN868351), Ovar-I*2E (LN868352), Ovar-I*2F (LN868353)	Ovar-DRB1*1201, Ovar-DRB1*0201	[39]
4247	Ovar-I*2G (LN868354), Ovar-I*2H (LN868355), Ovar-I*2I (LN868356), Ovar-I*2J (LN868357)	Ovar-DRB1*1102, Ovar-DRB1*0702	[39]

^a^ Local name assigned for partial mRNA sequence.

**Table 2:** Candidate genes for antigen screening.

Gene product, size	TA orthologues, % ID	TP orthologues, % ID	Putative function of orthologues	GenBank accession no.	Reference
Tl1, 454 aa	TA17450, 305/532 (57.3%)	TP03_0849 (Tp1), 209/557 (37.5%)	Hypothetical protein	KT989585	[17, 19]
Tl2, 177 aa	TA19865 (Ta2), 149/178 (83.7%)	TP01_0056 (Tp2), 109/177 (61.6%)	Surface protein d precursor	KT989586	[17, 19]
Tl3, 264 aa	TA06115, 236/265 (89.1%)	TP01_0868, 198/266 (74.4%)	Hypothetical protein	KT989587	[17] [19]
Tl5, 155 aa	TA14970 (Ta5), 154/155 (99.4%)	TP02_0767 (Tp5), 153/155 (98.7%)	Translation initiation factor eif-1A	KT989588	[17, 19]
Tl6, 277 aa	TA19320, 273/277 (98.6%)	TP01_0188, 274/277 (98.9%)	Prohibitin	KT989589	[17–19]
Tl7, 761 aa	TA12105, 706/723 (97.6%)	TP02_0244 (Tp7), 699/722 (96.8%)	Heat shock protein 90	KT989590	[17, 19]
Tl8, 413 a	TA11565, 382/413 (92.5%)	TP02_0140 (Tp8), 362/440 82.3%	Cysteine proteinase	KT989591	[17, 19]
Tl9, 311 aa	TA15705 (Ta9), 188/344 (54.7%)	TP02_0895 (Tp9), 169/366 (46.2%)	Secreted protein in infected cell cytoplasm	KT989592	[17, 19]
Tl10, 392 aa	TA10060, 377/448 (84.2%)	TP04_0772, 358/444 (80.6%)	Coronin	KT989593	[18, 19]
Tl12, 858 aa	TA08425, 666/894 (74.5%)	TP04_0437, 472/945 (49.9%)	microneme-rhoptry antigen (p104)	KT989594	[40]
Tl16, 275 aa	TA17315, 233/315 (74.0%)	TP04_0051, 176/488 (36.1%)	Surface protein precursor (TaSP or PIM)	KT989595	[19, 41]
Tl13245, 1628 aa	TA13245, 1410/1669 (84.5%)	TP02_0052, 495/1644 (30.1%), TP02_0051 736/1635 (45.0%)	Hypothetical protein	KT989596	[18]
Tl16020, 364 aa	TA16020, 277/370 (74.9%)	TP02_0952, 168/403 (41.7%)	Hypothetical protein	KT989597	This study

## Results

### Genotyping of MHC class I and II alleles

Class I and class II *DRB1* allele expression for each of the five immunized animals were determined by sequencing of cloned PCR products. All identified class I sequences represented novel alleles with the exception of allele U, which was identified in an earlier unpublished study (Table 1). Comparison of the predicted amino acid sequences of the novel alleles to the reference sequence N*00301 shows regions of polymorphism, particularly where residues were predicted to interact with peptides (S1 Fig). Class II sequence based genotyping identified a new *DRB1* allele (DRB1*1802) in animal 1263 and previously identified *DRB1* alleles in the other animals; none of the alleles were shared between the five animals (Table 1).

### Cytotoxic activity of CD8^+^ T cells from immunized sheep

**Fig 1:**
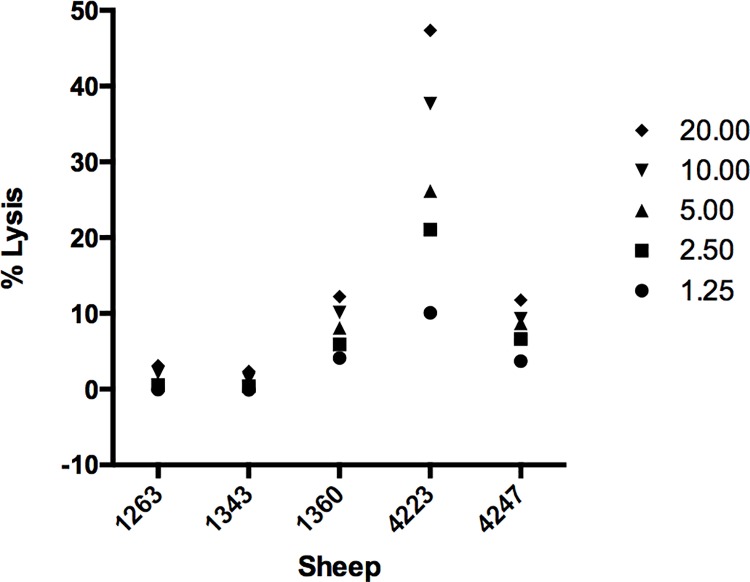
Cytotoxic activity of CD8^+^ T cells from immunized sheep measured by indium oxine release assays. Effector cells were stimulated twice with irradiated *T*. *lestoquardi*-infected cell lines and mixed with indium oxine labeled target *T*. *lestoquardi*-infected cells at indicated effector: target ratios.

**Fig 2:**
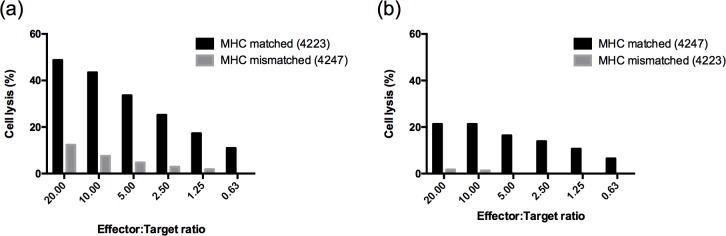
MHC-specific cytotoxicity of stimulated PBMC. **All sheep samples were assayed similarly and two representative data are shown.** (a) Sheep 4223 effectors lysed autologous infected cells more effectively than class I MHC-mismatched infected cells from Sheep 4247 at all effector: target ratios. (b) Sheep 4247 effectors lysed autologous infected cells, but not class I MHC mismatched infected cells from Sheep 4223.

CD8^+^ T cell responses of sheep immunized with live parasites were examined by *in vitro* stimulation of PBMC with irradiated autologous parasitized cells and testing the responding cells in a cytotoxicity assay using the same infected cells as targets. CD8^+^ T cell lines were established from all five immunized sheep following two or three *in vitro* antigen stimulations and then enrichment for CD8^+^ T cells by complement-mediated lysis of CD4 T cells; CD8^+^ T cells were the predominant cell type (66.95 ± 0.014%) in these lines (S2 Fig). CD8^+^ T cells from each immunized animal were then tested for cytotoxicity against autologous *T*. *lestoquardi*-infected cells in a cytotoxicity assay. The CD8^+^ T cell lines exhibited variable levels of killing; the line from one animal gave a maximal killing of 47% and two lines gave lower levels of lysis (4–12%), while lysis by the remaining two lines was not significantly above background (Fig 1). Stimulated PBMC showed MHC-restricted cytotoxicity when assayed with autologous and unrelated infected cells (Fig 2).

### Screening for *T*. *lestoquardi* antigens

A series of 13 *T*. *lestoquardi* candidate antigens were chosen to screen for recognition by the parasite-specific CD8^+^ T cell lines. They were selected based on orthology with *T*. *parva* and *T*. *annulata* antigens that were shown previously to be recognised by CD8^+^ T cells (Table 2), thus conforming to the premise that antigen recognition of *T*. *lestoquardi* in sheep is similar to that of *T*. *annulata and T*. *parva* in cattle. DNA sequences of the respective genes were obtained from a draft genome assembly of *T*. *lestoquardi* Lahr strain (W. Weir, unpublished data) and genes were obtained by PCR and sub-cloning the amplicons in recombinant plasmids.

Primary autologous fibroblast cell lines derived from the five sheep were transfected with recombinant plasmids incorporating each of the *T*. *lestoquardi* genes and screened for recognition by CD8^+^ T cells from the corresponding animal. Detection of a C-terminal V5 tag by immunofluorescence staining confirmed successful transfection of cells at 24 h and 48 h (S3 Fig).

**Fig 3:**
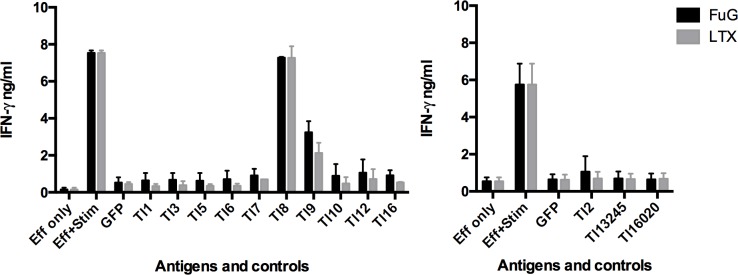
CD8^+^ T cell response to 13 *T*. *lestoquardi* antigens measured by IFNγ ELISA. Sheep 4247 fibroblasts were transfected with expression constructs of candidate antigen genes using either Lipofectamine LTX or Fugene transfection reagents for 48 h before the addition of effector cells for 72 h and IFNγ ELISA. The antigens were tested separately and data are presented as mean ± SD of biological repeats (n = 3).

Measurement of IFNγ release by antigen-stimulated CD8^+^ T cells demonstrated that one of five animals (Sheep 4247) responded to two *T*. *lestoquardi* antigens, Tl8 and Tl9 (Fig 3). There was no detectable response to any other antigen by T cell lines from this sheep or the other 4 sheep (data not shown). The CD8^+^ T cell screens included transfection using two different reagents–Lipofectamine LTX and Fugene. Although transfection efficacy was not assessed for Fugene, both reagents yielded similar positive results (Fig 3).

### Identification of CD8^+^ T cell epitopes

**Fig 4:**
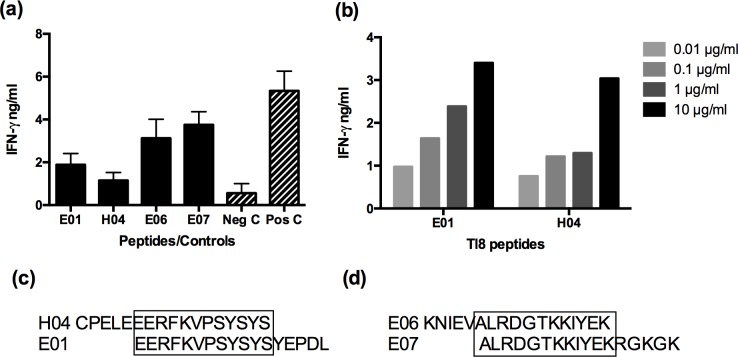
Tl8 and Tl9 peptide screen for CTL epitopes. (a) Peptides positively identified from peptide pools were incubated at 1 μg ml-1 with fibroblasts and assayed for IFNγ production by autologous stimulated effector cells. *T*. *lestoquardi*-infected cells incubated with effectors served as a positive control and effectors only served as a negative control. (b) Tl8 peptides were titrated and assayed for IFNγ production by autologous stimulated effector cells, which increased with increasing concentrations of peptides. (c) Overlapping peptide sequence of H04 and E01 of Tl8. (d) Overlapping peptide sequence of E06 and E07 of Tl9.

CD8^+^ T cells from sheep 4247, which recognised the Tl8 and Tl9 antigens, were tested for recognition of overlapping peptides spanning the full length of each protein. Positive results were obtained with two contiguous overlapping peptides from each antigen. These were peptides E01 and H04 of Tl8, and peptides E06 and E07 of Tl9 (Fig 4A). However, the IFNγ production responses detected with the Tl8 peptides at a concentration of 1 μg ml^-1^ were relatively weak. Further titration of the Tl8 peptides (at concentrations of 0.01–10 μg ml^-1^) (Fig 4B) demonstrated that a concentration of 10 μg ml^-1^ of Tl8 peptide was required to obtain an equivalent IFNγ response induced by the Tl9 peptides at a concentration of 1 μg ml^-1^. The overlapping peptide sequence of E01 and H04 of Tl8 was EERFKVPSYSYS, which corresponded to amino acid residues 241–252 (Fig 4C). The overlapping peptide sequence of E06 and E07 of Tl9 was ALRDGTKKIYEK, which corresponded to amino acid residues 271–282 (Fig 4D).

**Fig 5:**
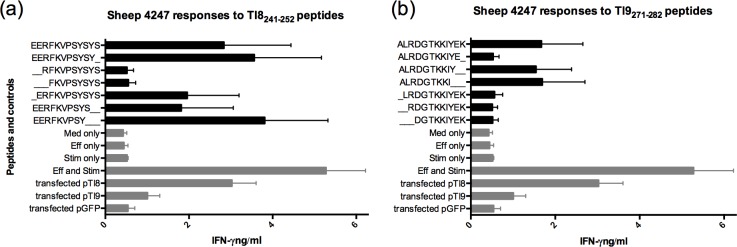
IFNγ **response of antigenic epitopes of (a) Tl8**_**241-252**_ **(EERFKVPSYSYS) and (b) Tl9**_**271-282**_ **(ALRDGTKKIYEK).** Different peptide sequence combinations ranging from 9-mer to 12-mer at 1 μg ml-1 were incubated for 24 h with fibroblasts before adding effector cells for 72 h. Cell supernatants were harvested for IFNγ assay (n = 2).

In order to identify the definitive epitopes within the Tl8_241-252_ (EERFKVPSYSYS) and Tl9_271-282_ (ALRDGTKKIYEK) sequences, a series of truncated peptides ranging from 9 to 12 amino acids in length were tested for recognition by the respective CD8 T cell lines (Fig 5). For the Tl8_241-252_, removing the first N-terminal Glu residue reduced IFNγ response, but removing the first two N-terminal Glu residues completely abolished the response (Fig 5A). A Tyr residue appears to be equally important at the C-terminus, as truncated peptides with this residue removed elicited a poor response, whereas truncated peptides with a C-terminal Tyr_249_ or Tyr_251_ stimulated the highest responses (Fig 5A). The 9-mer (Tl8_241-249_ EERFKVPSY) resulted in the highest responses for all three experimental repeats, followed in performance by the 11-mer (Tl8_241-251_ EERFKVPSYSY) and the 12-mer (Tl8_241-252_ EERFKVPSYSYS) (Fig 5A). For Tl9, a C-terminal truncated 9-mer peptide was recognised as effectively as the full length 12 mer (Fig 5B). Removing one or more of the first 4 N-terminal residues (Ala, Lys, Try, Ileu) resulted in a complete lack of T cell recognition (Fig 5B). Hence the shortest antigenic peptide to induce a strong IFNγ response was the 9-mer Tl9_271-279_ ALRDGTKKI (Fig 5B).

A comparison of the amino acid sequence of Tl8 to that of the *T*. *annulata* and *T*. *parva* orthologues Ta8 and Tp8 showed 87.65% of residues were similar. Tl8_241-249_ EERFKVPSY did not overlap with the previously identified epitope of Tp8_379-387_ CGAELNHFL [29] (S4 Fig). The sequence of the corresponding 9 amino acid regions in Ta8 and Tp8 each differed by only one amino acid residue from the Tl8 epitope sequence (S4A Fig). A similar comparison of the amino acid sequences of Tl9 to Ta9 and Tp9 revealed only 51.9% similarity, although the N-terminal predicted signal peptide was highly conserved (S4B Fig). The latter is consistent with previous comparisons of Ta9 and Tp9 [19]. The positions of the epitope in the Tl9 protein differed from those of previously identified epitopes in the *T*. *annulata* and *T*. *parva* orthologues (S4 Fig). In contrast to Tl8, the amino acid sequences of the corresponding 9 amino acid regions in Ta9 and Tp9 differed at 5 and 6 amino acid positions respectively from the Tl9 sequence.

## Discussion

A subunit vaccine against *T*. *lestoquardi* is an attractive alternative strategy to live attenuated vaccines; however, there is limited available data on *T*. *lestoquardi* antigens and the immune responses against them. This is the first report of CD8^+^ T cells from immunized sheep having cytotoxic activity against *T*. *lestoquardi*-infected lymphocytes and identification of potential *T*. *lestoquardi* antigens that can be recognised by this CD8^+^ T cell response. In addition, the results confirm that orthologues of antigens that are recognised by the bovine CD8^+^ T cell response are also recognised by the ovine immune response, although the identified epitopes may vary.

While initial cytotoxicity assays lacked a control to confirm specificity against parasite-infected cells, subsequent IFNγ assays were carried out with enriched CD8^+^ T cells and included uninfected fibroblasts, confirming parasite-specific responses mediated by CD8^+^ T cells. The relatively low cytotoxic activity of the sheep 4247 CD8^+^ T cell line nonetheless having a robust IFNγ response to Tl8 and Tl9 indicates that levels of cytotoxicity do not necessarily reflect the CD8 T cell IFNγ response. This is similar to observations with CD8^+^ T cell lines generated from cattle immunized against *T*. *annulata*, where there was variability in cytotoxicity but a consistent and specific IFNγ response to antigens [30].

Tl8 is predicted as a cysteine proteinase and Tl9 has no known predicted function but is unique to transforming Theileria species; a Ta9/Tp9 orthologue was absent in the non-transforming species, *T*. *orientalis* [42]. Sequence analysis by SignalP 4.1 [43] did not predict a signal peptide in Tl8 or Ta8, while Tp8 has a predicted signal anchor peptide [17], suggesting that Tl8 and Ta8 may not be secreted proteins, while Tp8 may be a membrane protein. In contrast, Tl9 is predicted to contain a signal peptide, similar to Ta9 and Tp9. Ta9 has been reported to be secreted into the cytoplasm of macroschizont infected cells [19]. Tp8, Tp9 and Ta9 have been shown to be antigenic in cattle [17, 19, 29].

The detection of T cell responses to two of only thirteen candidate antigens, in one of five immunized sheep is a reasonable detection rate relative to previous antigen screening studies for *T*. *parva* and *T*. *annulata* [17, 19]. The data confirms that some parasite gene products serve as antigens in several Theileria species, and that *T*. *lestoquardi* antigens are recognized by CD8^+^ T cells from different animals in a preferential manner, as is the case for *T*. *parva* and *T*. *annulata* in cattle [19, 29, 31]. Previous studies of *T*. *parva* indicated that the latter reflects an effect of MHC type on antigenic dominance. In this regard, the MHC type of sheep 4247 differed from that of the other sheep examined. A high peptide concentration was required to detect the Tl8 epitope, which was likely due to peptide insolubility issues rather than a lack of reactivity *per se*, for the following reasons: a) RPMI was used for resuspension of peptide and precipitates were observed; b) specific T cells were present, as pTl8 transfected fibroblast controls produced high levels of IFNγ (5.3 ± 1.4 ng ml^-1^ for LTX and 3.9 ± 0.9 ng ml^-1^ for Fugene); c) BLASTP of the Tl8 epitope sequence against the translated sequence of the *T*. *lestoquardi* genome did not result in any high-scoring matches (other than Tl8), arguing against the possibility that the detected Tl8 response represented cross-reactivity with a true epitope in another protein [44].

For both Tl8 and Tl9, we determined that 9-mer peptides were optimal for inducing an IFNγ response. This length is consistent with *T*. *parva* antigenic peptides derived from Tp2, Tp4, Tp5, Tp7 and Tp8 in cattle, which are all 9-mers [29]. However, the peptide sequences did not overlap with previously identified antigenic epitope sites in the Tp8 and Ta9 proteins. This may be due to differences in the peptide binding sites of MHC I molecules of sheep and cattle, other antigen-dependent factors such as peptide conformations and unidentified epitopes, or a combination of unidentified MHC I molecules and epitopes. In this regard, prediction of *T*. *parva* and *T*. *annulata* epitopes binding to bovine MHC I (BoLA) molecules by *NetMHCpan* has generated a greater number of epitopes and binding BoLA molecules than peptide mapping or truncation assays [45, 46]. This tool could be useful for the prediction of additional putative *T*. *lestoquardi* epitopes when more data on ovine MHC I alleles becomes available.

Analysis of the MHC class I diversity in sheep has been limited to alleles from Scottish Blackface [39] and French Prealpe breeds (K. Ballingall, unpublished data). Therefore, it was unsurprising that most alleles identified in the Swaledale/Leicester cross sheep used in this study were novel and diverse. However, it is of interest that alleles 2G, H, I, J from the responding sheep 4247 have a unique polymorphic cluster corresponding to changes Phe_113_ and Met_114_ of the reference sequence N*00301; Met_114_ was predicted to bind peptides [33]. Allele 2K also possessed this unique cluster, although it is one of three alleles identified in an unresponsive sheep.

To summarize, we were able to identify two *T*. *lestoquardi* proteins that are recognised by a CD8^+^ T cell line established *in vitro* from an MHC I defined immune sheep, indication that they are involved in potentially protective T cells response *in vivo* against *T*. *lestoquardi*-infected leukocytes. Furthermore, we were able to deduce the minimal length peptides required for recognition of these antigens by a CD8^+^ T cells response. More comprehensive antigen screens and additional studies are necessary to determine the genetic diversity of both parasite and host, and to test the ability of identified proteins to induce protection against *T*. *lestoquardi* challenge. While the use of an attenuated *T*. *lestoquardi*-infected cell line has shown protection in one study [12], a more targeted approach to vaccination, ideally utilising antigens conserved between different strains of *T*. *lestoquardi*, has practical advantages in terms of safety, production, storage and administration.

## Supporting Information

S1 Fig Alignment of translated allele sequences and comparison to an MHC class I sequence N*00301 (AJ874673). Conflicting residues are shown, consensus residues are indicated by dots, gaps are indicated by dashes, and MHC class I residues previously predicted to interact with peptides presented to T cells are indicated by asterisks. Residues are numbered according to N*00301 sequence.
(TIFF)

S2 Fig CD8^+^ T cells was the predominant cell population in PBMCs of sheep immune to *T*. *lestoquardi* after three stimulations. (TIF)

S3 Fig Expression of *T*. *lestoquardi* genes in transfected fibroblasts of either sheep 1263 or 1360. Expression was detected by antibodies against the C-terminal V5 tag (green). Cells were counterstained with DAPI (blue).
(TIF)

S4 Fig **Sequence comparisons of (a) Tl8**_**241-249**_ **EERFKVPSY and (b) Tl9**_**271-279**_ **ALRDGTKKI to *T*. *annulata* and *T*. *parva* orthologues.** Conflicting residues are in red, antigenic epitopes identified in this study and in previous studies (Tp8_379-387_ CGAELNHFL, Ta9_40-49_ QRSPMFEGTL, and Ta9_64-72_ SKFPKMRMG) are boxed in purple, predicted signal peptide sequences are indicated, and conserved residues within an epitope region are indicated by asterisks.
(TIFF)

S1 Table Primers used for cDNA amplification and cloning. (DOCX)
