# Supplementary figures and images for: Identification of Theileria lestoquardi Antigens Recognized by CD8+ T Cells
Source: PLoS One. 2016 Sep 9;11(9):e0162571. doi: 10.1371/journal.pone.0162571 (PMC5017765; doi:10.1371/journal.pone.0162571)

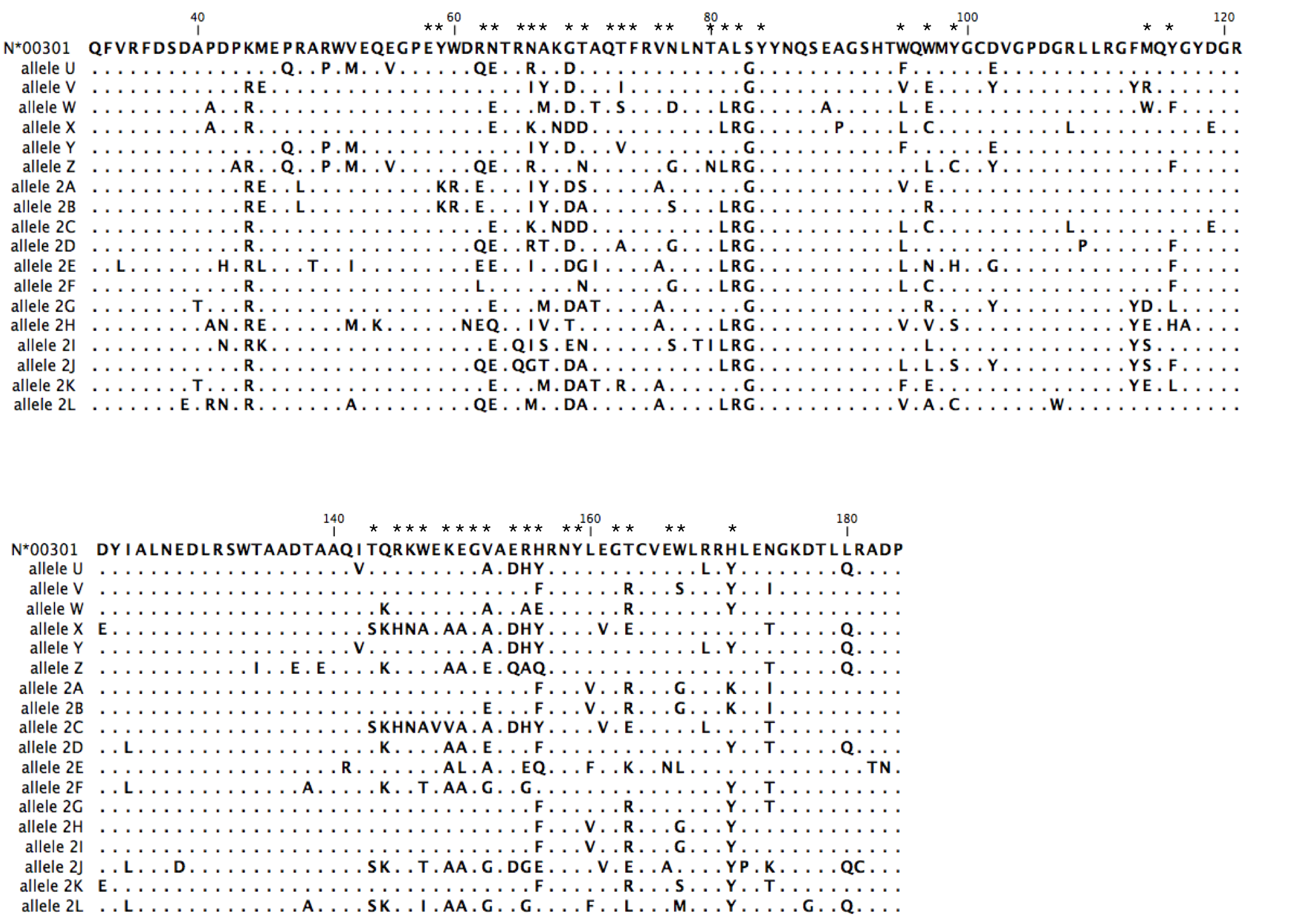

Supplement: S1 Fig — Conflicting residues are shown, consensus residues are indicated by dots, gaps are indicated by dashes, and MHC class I residues previously predicted to interact with peptides presented to T cells are indicated by asterisks. Residues are numbered according to N*00301 sequence. (TIFF) [file pone.0162571.s001.tiff]

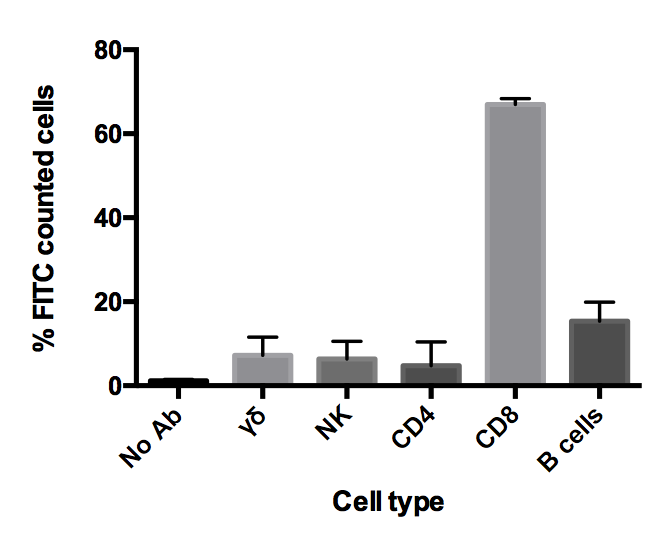

Supplement: S2 Fig — (TIF) [file pone.0162571.s002.tif]

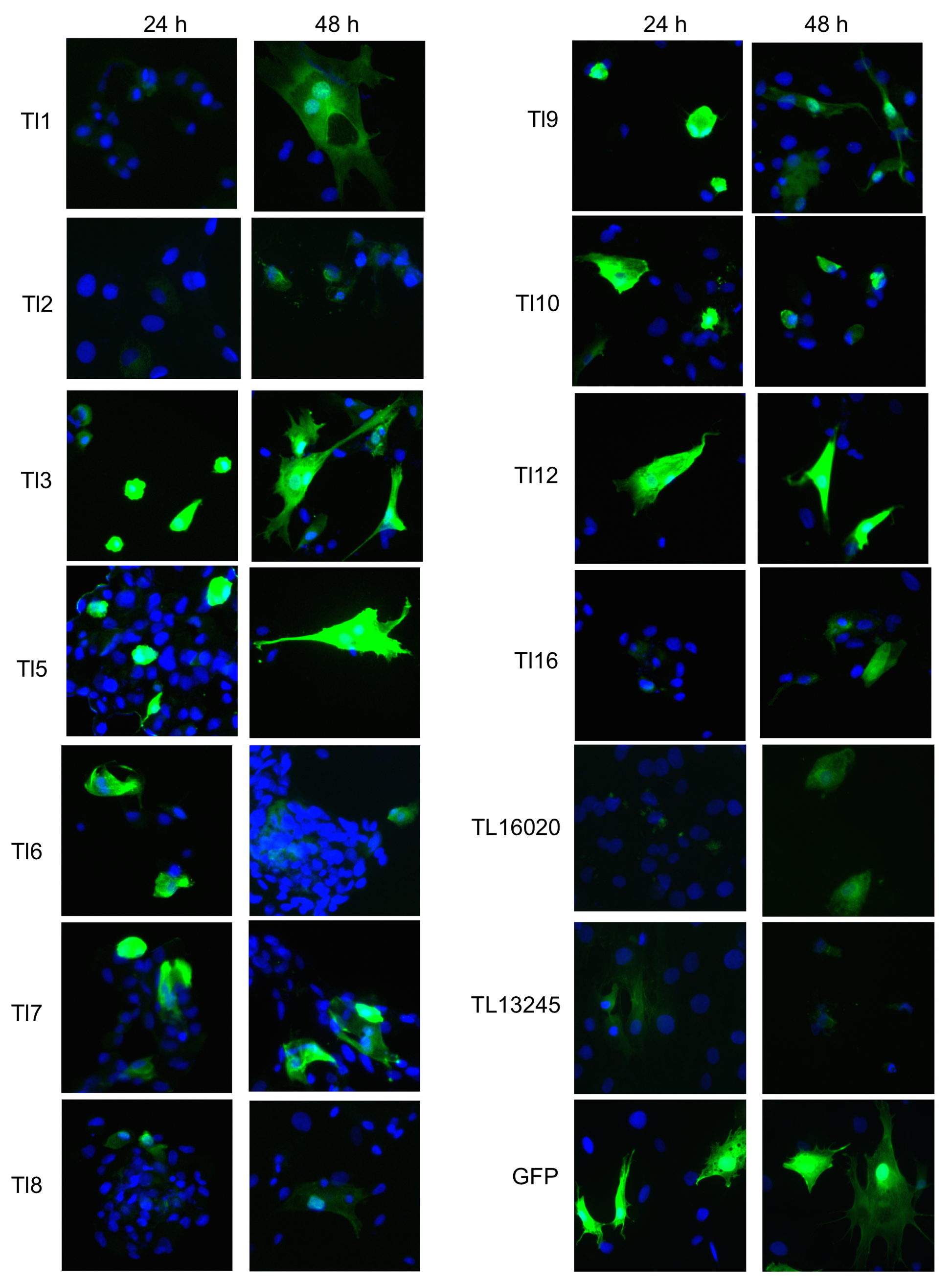

Supplement: S3 Fig — Expression was detected by antibodies against the C-terminal V5 tag (green). Cells were counterstained with DAPI (blue). (TIF) [file pone.0162571.s003.tif]

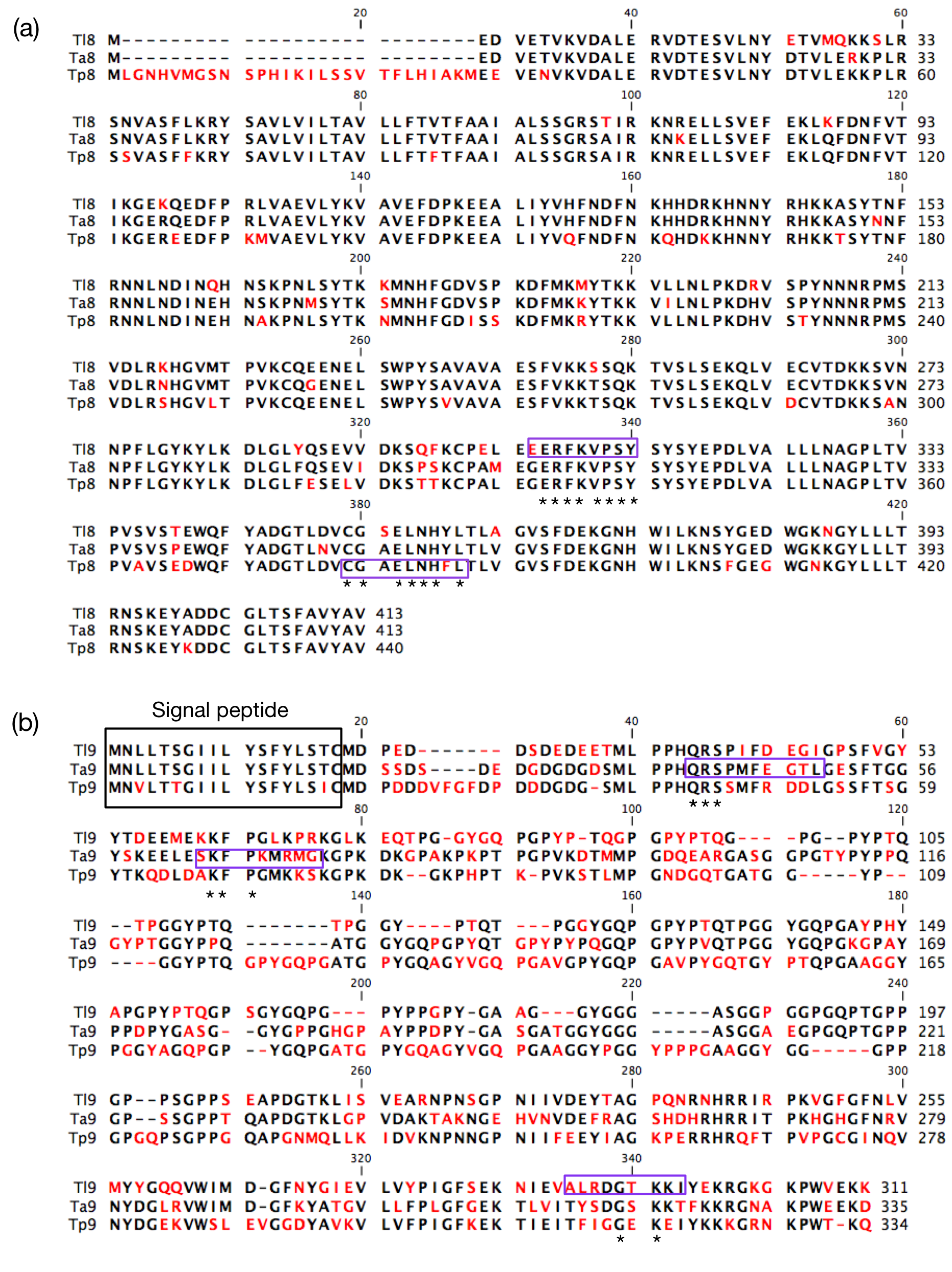

Supplement: S4 Fig — Sequence comparisons of (a) Tl8241-249 EERFKVPSY and (b) Tl9271-279 ALRDGTKKI to T. annulata and T. parva orthologues. Conflicting residues are in red, antigenic epitopes identified in this study and in previous studies (Tp8379-387 CGAELNHFL, Ta940-49 QRSPMFEGTL, and Ta964-72 SKFPKMRMG) are boxed in purple, predicted signal peptide sequences are indicated, and conserved residues within an epitope region are indicated by asterisks. (TIFF) [file pone.0162571.s004.tiff]
