# Supplementary material for: Identification of Theileria lestoquardi Antigens Recognized by CD8+ T Cells
Source: PLoS One. 2016 Sep 9;11(9):e0162571. doi: 10.1371/journal.pone.0162571 (PMC5017765; doi:10.1371/journal.pone.0162571)
Supplement: S1 Table — (DOCX) [file pone.0162571.s005.docx]

Table S1. Primers used for cDNA amplification and cloning

| Primer | Sequence | Purpose | Amplicon size |
| --- | --- | --- | --- |
| T.1.1F_EcoRV | atatGATATC**atg**caaaaggaacagttagaatc | Amplification of Tl1 from cDNA | 1302 bp |
| T.l.1R_XhoI | atatCTCGAGgtttttgatgtagatttg |  |  |
| T.l.3F_XbaI | atatTCTAGA**atg**ctcaggcgtagttctcc | Amplification of Tl3 from cDNA | 729 bp |
| T.l.3R_XhoI | atatCTCGAGtgattttttastcttcttc |  |  |
| T.l.5F_EcoRV | atatGATATC**atg**ccgaaaaataaaggt | Amplification of Tl5 from cDNA | 465 bp |
| T.l.5R_XhoI | atatCTCGAGcaaatcgtcgatgtcgaaatc |  |  |
| T.l.6F_EcoRV | atatGATATC**atg**tgggtggtcaattccag | Amplification of Tl6 from cDNA | 753 bp |
| T.l.6R_XhoI | atatCTCGAGtttatcagttgagagtagaag |  |  |
| T.l.7F_XbaI | atatTCTAGA**atg**acatcaaacgaggagacacc | Amplification of Tl7 from cDNA | 2148 bp |
| T.l.7R_BamHI | atatGGATCCgtcaacttcctccattttggag |  |  |
| T.l.8F_EcoRV | atatGATATC**atg**tcatcaggcagaagcac | Amplification of Tl8 from cDNA | 1047 bp |
| T.l.8R_XhoI | atatCTCGAGtactgcgtatactgcaaag |  |  |
| T.l.9F_EcoRV | atatGATATC**atg**gatcctgaagatgattctg | Amplification of Tl9 from cDNA | 879 bp |
| T.l.9R_XhoI | atatCTCGAGttttttttctacccatggtttgcc |  |  |
| T.l.10F_XbaI | atatTCTAGA**atg**gcgataattgacattaacaac | Amplification of Tl10 from cDNA | 1176 bp |
| T.l.10R_XhoI | atatCTCGAGcgtcttgattgttttaa |  |  |
| T.l.12F_EcoRV | atatGATATC**atg**gacgacctagtcatgagt | Amplification of Tl12 from gDNA | 2454 bp |
| T.l.12R_XhoI | atatCTCGAGtttctttggttttggtgtct |  |  |
| T.l.16F_EcoRI | atatGAATTC**atg**aaattcttctacctttttgttc | Amplification of Tl16 from cDNA | 825 bp |
| T.l.16R_XhoI | atatCTCGAGacaacaatcttcgttaatgc |  |  |
| Tl2_F | gctacaccctatgattcccc | Amplification of Tl2 from cDNA | 719 bp |
| Tl2_R | ttaacaccaaagcctgaagac |  |  |
| Tl13245_F | atgaaaaaatttcgttctcca | Amplification of TL13245 from cDNA | 4800bp |
| Tl13245_R2 | ttaatgtgttccttcggca |  |  |
| Tl16020_F | atgaaatttctctctaagatactattactc | Amplification of TL16020 from cDNA | 1094bp |
| Tl16020_R | tcattccgtatcaatttcttc |  |  |
| pJET1.2_F | cgactcactatagggagagcggc | Sequencing of cloned inserts in pJET | 119bp* |
| pJET1.2_R | aagaacatcgattttccatggcag |  |  |
| Tl2_SLIC_F | ttctctagagatatcatgaaattgaccgctggatt | Sequence and ligation independent cloning (SLIC) of TQ2 into pmax | 572 bp |
| Tl2_SLIC_R | aggcttaccctcgagtgaaccccccgaagcttc |  |  |
| Tl13245_LIC_F2 | gaattctctagagatatgaaaaaatttcgttctcca | SLIC of TL13245 into pmax | 4900bp |
| Tl13245_LIC_R2 | ggcttaccctcgagtttaatgtgttccttcggcat |  |  |
| Tl16020_FEcoRV | tactatgatatcatgaaatttctctctaagatact | Restriction-ligation cloning of TL16020 into pmax | 1114 bp |
| Tl16020_R2XhoI | gtctcgagttccgtatcaatttcttca |  |  |
| pmax_EcoRV | gacatgatatctctagagaattcaagcttgtttaaac | Inverse PCR to linearise pmax for SLIC | 2900bp |
| pmax_XhoI | agtactcgagggtaagcctatccct |  |  |
| Pmax_F | gaaactgggcttgtc | Sequencing of cloned inserts in pmax | 308 bp* |
| Pmax_R | ttaacttgtttattg |  |  |
| CMV-FSG | tagtgaaccgtcagatcac | Sequencing of cloned inserts in pmax | 397bp* |
| SV40pA-R | gcaatagcatcacaaatttc |  |  |

*In the absence of an insert
